# Screening for distress in patients with intracranial tumors during the first 6 months after diagnosis using self-reporting instruments and an expert rating scale (the basic documentation for psycho-oncology short form – PO-Bado SF)

**DOI:** 10.18632/oncotarget.25763

**Published:** 2018-07-24

**Authors:** Mirjam Renovanz, Helena Tsakmaklis, Sari Soebianto, Isabell Neppel, Minou Nadji-Ohl, Manfred Beutel, Andreas Werner, Florian Ringel, Anne-Katrin Hickmann

**Affiliations:** ^1^ Department of Neurosurgery, University Medical Center, Johannes-Gutenberg-University of Mainz, Mainz, Germany; ^2^ Department of Neurosurgery, Klinikum Stuttgart, Katharinenhospital, Stuttgart, Germany; ^3^ Department of Spine Surgery and Neurosurgery, Schulthess Klinik, Zürich, Switzerland; ^4^ Department of Psychosomatic Medicine and Psychotherapy, University Medical Center, Johannes-Gutenberg-University of Mainz, Mainz, Germany

**Keywords:** brain tumor patients, psychosocial screening, Po-BADO, external assessment, neuro-oncology

## Abstract

**Objective:**

Psychosocial screening in brain tumor patients is of high importance. We applied The Basic Documentation for Psycho-Oncology Short Form (PO-Bado SF) in primary brain tumor patients and patients with metastasis. The aim was to evaluating consistency between physicians' perception and the results of the patients' self-assessment.

**Materials and Methods:**

140 patients with first diagnosis of a brain tumor were screened during their hospital stay (t1) using Distress Thermometer (DT) and Hornheide Screening Instrument (HSI), health-related quality of life was assessed by EORTC QLQ-C30 + BN20. After 3 (t2) and 6 months (t3), patients were re-evaluated. Attending neuro-oncologists completed the PO-Bado SF at all three time points (cut-off for being in need for support >8).

**Results:**

At t1, the mean of the PO-Bado SF total score was 7.71 (SD = 4.08), at t2 8.22 (SD = 5.40) and at t3 7.62 (SD = 5.72).

The proportion of patients reaching a total score >8 was at t1: 43%, at t2: 41% and at t3: 47% (t1–3). Discrimination of PO-Bado SF total score, between patients in (DT ≥6) and those not in distress was more sensitive (cut-off 8.5, AUC 0.772, sens. 71.3%, spec. 67.6%) than discrimination compared to the HIS (cut-off 9.5, AUC 0.779, sens. 65.1%, spec. 77.7%). Higher PO-Bado-SF total score correlated with higher DT scores (*r* = 0.6, *p* < 0.0001) and lower EORTC GHS scores (*r* = −0.55, *p* < 0.0001).

**Conclusion:**

Physicians' perception according to PO-Bado SF provides a different measure for psychosocial burden in patients with brain tumors, however does not completely reflect patients' wishes.

## INTRODUCTION

The diagnosis of an intracranial tumor causes mental strain and distress for patients regardless of the tumor entity [1–4]. Recently, a meta-analysis of Huang *et al.* including 37 studies with *n* = 4518 brain tumor patients showed a mean prevalence of depressive symptoms and depression in 21.7% of patients, which was higher compared to the normal population [5]. This holds true also for patients without a dismal prognosis: Preoperative symptoms of depression and anxiety seem to be associated with an elevated 5-year overall mortality risk in meningioma patients (Bunevicius *et al.* [6]). Therefore, adequate and timely assessment of psychosocial burden is relevant in patients with intracranial tumors regardless of its entity. During recent years, screening for distress have been developed in order to be part of the assessment in clinical routine – however the assessment and interpretation of results remains challenging [7, 8]. Mostly, self-reporting questionnaires or screening instruments are used – e.g. the Distress Thermometer - with the advantages of objectivity in scoring and brevity as well as direct assessment of the patients’ perspective [9, 10].

However, feasibility and acceptance by both patients and health professionals remains suboptimal [11]. Due to neurocognitive impairment of patients with intracranial lesions and/or restricted ability to undergo screening procedures for physical and psychological reasons the distress of certain patients may remain unrecognized [12–14].

Therefore, the assessment of psychosocial distress by interviewers (ClinRO) may provide an important addition to screening for psychosocial burden in patients who are unwilling or unable to fill in self-report questionnaire. The Basic Documentation for Psycho-Oncology Short Form (PO-Bado SF) is a clinician-administered instrument to guide professionals in a focused and structured psycho-oncological assessment developed from The Basic Documentation for Psycho-Oncology [15]. It has been used with different cancer populations [15–19] and consists of an expert rating scale with six items that are rated on a five-point Likert scale (0 = not at all, to 4 = very much) and a short structured interview. A total score of 8 or greater on the PO-Bado SF is recommended to recognize patients with clinically relevant distress [17], however it has not been validated in brain tumor patients so far. Marten-Mittag *et al.* conducted an analysis comparing – inter alia - the Distress Thermometer (DT) values of 1551 cancer patients (clinically relevant distress: DT > 4) and the results of PO-Bado SF results scored by physicians and found a total score of 9 or greater to be optimal [18]. However, in the patient sample there were rare brain tumor patients. Therefore, we were interested in analyzing the instrument especially in brain tumor patients with regard to its feasibility. As brain tumor patients may not always be able to perform self-assessment it would be helpful to apply the PO-Bado SF as a clinician reported outcome (ClinRO) instead of PRO to assess distress in brain tumor patients, which is of high clinical relevance.

In our prospective observational study we evaluated the psychosocial burden during the early disease trajectory of brain tumor patients six months after first diagnosis by self-reporting questionnaires but also by the PO-Bado-SF as an expert rating scale.

We investigated 1) the comparability between physicians’ perception of the patients’ burden and the results of the patients’ self-assessment, 2) if the PO-Bado SF total score cutoff described in the literature can be applied to brain tumor patients as well and 3) the capability of the PO-Bado SF global burden (GB) as single item to reliably identify need for psychosocial support/distress.

## RESULTS

### Patients

**Figure 1:**
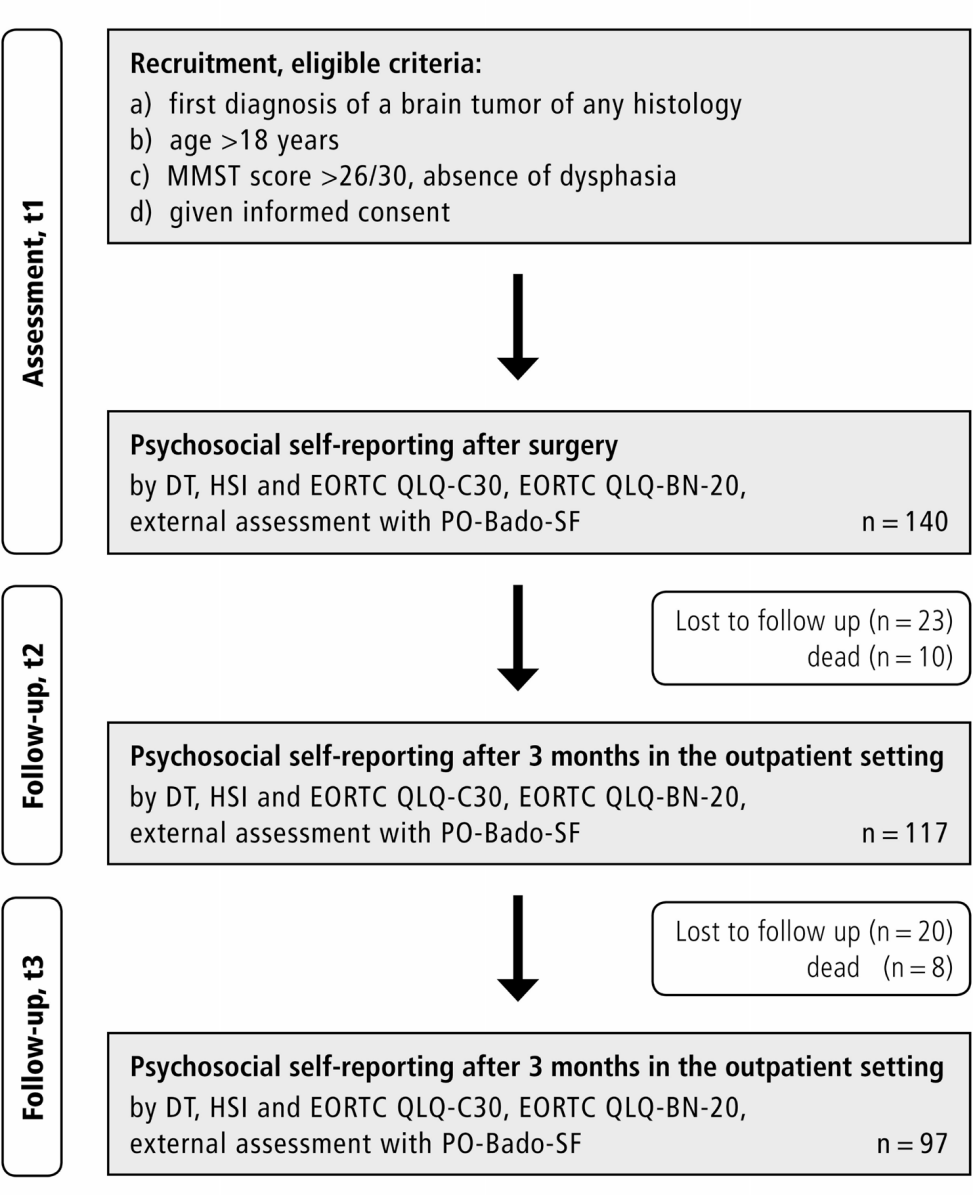
Setting and course of the study

Data of 140 patients recruited between September 2012 and September 2014 was analyzed with a slight female predominance in both centers (female patients *n =* 76, 54%) as shown in Figure 1. The mean age of patients was 56 years (SD = 12 years). No clinical impairment was observed after surgery, the majority of patients had a KPS of ≥ 70 pre- and postoperatively with score being significantly more often ≥ 70 at the community hospital (KPS ≥ 70: preoperatively *n =* 122, 87% and postoperatively *n =* 128, 92%). Most of the patients suffered from malignant gliomas (37%) and meningiomas (31%). Metastasis from solid tumors occurred in 22%. Most of the tumors were located in the frontal lobe (*n =* 41, 33%). In the majority of cases a gross total resection (GTR) could be achieved (*n =* 103, 75%). All patient and tumor characteristics as well as demographic data are displayed in Table 1. Between t1 and t2 *n =* 23 patients and between t2 and t3 further *n =* 20 dropped out, *n =* 10 died until t2 and *n =* 8 until t3. At t2 *n =* 9 and at t3 *n =* 4 were excluded due to incompliance to assessment (did not fill in the questionnaires as scheduled).

**Table 1:** Patients’ characteristics of all included subjects

	All	University	Community	*p*-value
Age (years)	55.9 ± 11.6	58.1 ± 11.1	54.3 ± 11.8	0.068^#^
Gender				0.85^*^
Male	64 (45.7)	28 (46.7)	36 (45.0)	
Female	76 (54.3)	32 (53.3)	44 (55.0)	
Karnofsky Performance Scale				
≥70 preoperatively	122 (87.1)	42 (70)	80 (100)	<0.0001^*^
≥70 postoperatively	128 (92.1)	48 (81.4)	80 (100)	<0.0001^*^
Tumor-Entity				0.134^*^
Metastasis	30 (21.9)	16 (28.1)	14 (17.5)	
Astrocytic Tumor	50 (36.5)	23 (40.4)	27 (33.8)	
Meningeoma	42 (30.7)	15 (26.3)	27 (33.8)	
Other	15 (10.9)	3 (5.3)	12 (15)	
Tumor Localisation				0.521^*^
Frontal	41 (33.1)	18 (31.0)	23 (34.5)	
Temporal	20 (16.1)	12 (20.7)	8 (12.1)	
Parietal	16 (12.9)	7 (12.1)	9 (13.6)	
Occipital	13 (10.5)	4 (6.9)	9 (13.6)	
Infratentorial	21 (16.9)	9 (15.5)	12 (18.2)	
Multiple	13 (10.5)	8 (13.8)	5 (7.6)	
Extend of Resection				0.046^*^
GTR	103 (74.6)	39 (66.1)	64 (81.0)	
No GTR	35 (25.4)	20 (31.7)	15 (19.0)	

*p*-values for distribution between groups (University vs. Community), ^#^Mann-Whitney-*U*-Test, ^*^Pearson-Chi-Square Test.

### Results of PO-Bado SF at t1, t2 and t3

The PO-Bado SF interview was conducted in *n =* 139 participating patients at t1, resp. *n =* 117 at t2 and *n =* 96 at t3 (patient drop outs: t2 *n =* 23 and at t3 further *n =* 20, reasons please see above). Mean time of PO-Bado SF assessment was 7.5 min (range 5–18.5 min). At t1, the mean of the PO-Bado SF total score was 7.71 (SD = 4.08, range 0–20), at t2 8.22 (SD = 5.40, range 0–22) and at t3 7.62 (SD = 5.72, range 0–23).

The proportion of patients reaching a total score ≥8 was at t1 *n =* 60 out of 139 (43%), at t2 *n =* 49 out of 117 (41%) and at t3 *n =* 45 out of 96 (47%).

Only *n* = 20 (14%) patients were estimated as being in need of support by the clinicians consistently at all three time points (total score ≥ 8) and *n =* 26 with complete FU were assessed by a total score ≥ 8 at t1 and t3 (Table 2 and Supplementary Table 1).

**Table 2:** Results of PO-Bado screening at all three measurements with regard to the total score, global burden and the subscales as well as the proportion in need of psychosocial intervention according to the cut-off > 8

Item	post-OP/t1 mean ± SD	3 months/t2 mean ± SD	6 months/t3 mean ± SD
PO-Bado-SF global burden	4.32 ± 2.00	4.06 ± 2.36	3.75 ± 2.55
PO-Bado-SF total score	7.71 ± 4.08	8.33 ± 5.40	7.62 ± 5.72
Fatigue/tiredness	1.23 ± 0.80	1.80 ± 1.27	1.55 ± 1.12
Mood swings/helplessness/vulnerability	1.23 ± 1.05	1.36 ± 1.01	1.22 ± 1.11
Anxiety/worries/tension	1.83 ± 1.08	1.50 ± 1.15	1.45 ± 1.21
Depression/grief	1.32 ± 1.08	1.11 ± 1.12	1.04 ± 1.07
Functional limitations in daily activities	1.33 ± 0.92	1.54 ± 1.13	1.29 ± 1.17
Other problems, e.g. social or family problems Proportion of patients with total score ≥ 8 (%)	0.83 ± 1.02 43	1.01 ± 1.01 41	1.07 ± 1.22 47

The item means range between the highest 1.80 (SD = 1.27) for “fatigue/tiredness” at t2 and the lowest 0.83 (SD = 1.2) for “other problems, e.g. social or family problems” also at t1.

Results are displayed in more detail in Table 2.

Significant differences between tumor entities were only seen for PO-Bado SF GB scores postoperatively and at t2 for PO-Bado SF total score by univariate explorative analyses. Complete results are shown in the Supplementary (Supplementary Table 2).

### The results of DT and HSI in (dis-) concordance with PO-Bado SF

At t1, DT identified *n =* 51 (36%) patients as being in need of support. After 3 and 6 months *n =* 29 (25%), resp. *n =* 22 (23%), scored ≥6 on the DT. With the HSI, comparable percentages of patients in need of psychosocial help were identified by the screening (t1: *n =* 41/29%, t2: *n =* 40/34%, t3: *n =* 31/32%).

DT-Scores improved over the course of the investigation with significant differences between assessments. The need for treatment based on the HSI was comparable between assessments.

PO-Bado SF showed only fair agreement with the screening results of DT and HSI regarding patients’ need for psychosocial intervention [20]: Regarding the DT, agreement with the ClinRO (PO Bado) was observed in 51–58% (κ = 0.315, *p* < 0.001), and regarding the HSI in 46–61% (κ = 0.352, *p* < 0.001) of the cases. Further, *n* = 34 (24%) patients requested psychological support after diagnosis was confirmed (t1) of which 19 (80%) were identified by physicians via PO-Bado-SF as being in need for support at the same time. After 3 resp. 6 months, *n* = 12 resp. *n* = 11 patients (10% resp. 11%) immediately asked for psychological help, by PO-Bado-SF *n* = 8 (67%) resp. *n* = 6 (54%) of these patients were detected as in need for support. Similarly, the DT results and HSI results were only partially in agreement. Further detailed information on DT and HSI results as well as agreement between results of external assessment and self-reporting questionnaires are shown in Table 3.

**Table 3:** Results of DT and HSI

***DT-Scores***			
	postop	3 months	6 months
Mean ± SD	4.9 ± 2.5	4.1 ± 2.6	3.5 ±2.5
Missing^#^ (*n*/%)	4/3	27/19	51/36
			
Agreement^*^ +/+ (*n*)	27	17	14
Agreement^*^ −/− (*n*)	53	47	35
Agreement^*^ all (*n*/%)	80/58	64/55	49/51
***HSI – need for treatment***			
	postop	3 months	6 months
Yes (*n*/%)	41/30	40/29	31/22
No (*n*/%)	98/70	72/51	63/45
Missing^#^ (*n*/%)	1/1	28/20	46/33
			
Agreement^*^ +/+ (*n*)	26	23	14
Agreement^*^ −/− (*n*)	59	42	30
Agreement^*^ all (*n*/%)	85/61	65/56	44/46
			
***DT and HSI agreement***			
	postop	3 months	6 months
Agreement +/+ (*n*)	25	17	15
Agreement −/− (*n*)	62	51	37
Agreement all (*n*/%)	87/62	68/58	52/54

Results according to patients’ self-assessment by DT and HSI in comparison to the external assessment results of PO-Bado.

^*^Agreement +/+: distress or need for psycho-oncological intervention according to Po-BADO and DT/HSI, Agreement −/−: no relevant distress and patient not need for need for psycho-oncological intervention according to Po-BADO and DT/HSI.

^#^All missings including drop-outs are described.

### PO-Bado SF total score cut-offs in our patient sample

Using the PO-Bado SF total score, discrimination between patients in and those not in distress (DT ≥ 6) showed a greater sensitivity (cut-off 8.5, AUC 0.772, sens. 71.3%, spec. 67.6%) than discrimination between patients in need of treatment according to the HSI, which however turned out to provide a better specificity (cut-off 9.5, AUC 0.779, sensitivity 65.1%, specificity 77.7%). More results of the receiver operator characteristic analyses utilizing PO-Bado-SF total score are displayed in Table 4.

**Table 4:** PO-Bado cut-off values indicating increased distress (DT ≥ 6) and need for treatment (HSI) (ROC Analysis)

**DT ≥ 6**				
	Cut-off	AUC	Sensitivity	Specificity
GB postop	4.5	0.731	64.6%	67.1%
GB 3 months	4.5	0.839	78.6%	75%
GB 6 months	4.5	0.883	81.0%	75.7%
GB all	4.5	0.806	72.2%	72.8%
Total score				
postop	9.5	0.765	56.3%	84.5%
Total score				
3 months	8.5	0.758	76.0%	68.3%
Total score				
6 months	7.5	0.847	90.5%	66.7%
Total score				
all	8.5	0.772	71.3%	67.6%
**HSI – need for treatment**				
	Cut-off	AUC	Sensitivity	Specificity
GB postop	5.5	0.772	59.0%	84.6%
GB 3 months	3.5	0.744	75.0%	57.7%
GB 6 months	3.5	0.749	76.7%	68.3%
GB all	3.5	0.749	81.7%	56.4%
Total score				
postop	9.5	0.787	66.7%	79.6%
Total score				
3 months	6.5	0.800	89.2%	60.9%
Total score				
6 months	7.5	0.749	67.7%	67.7%
Total score				
all	9.5	0.779	65.1%	77.7%

GB = PO-Bado Global Burden; Total score = PO-Bado SF total score

Cut-off values were selected according to best statistical balance between sensitivity and specificity (Akobeng *et al.* 2006).

### Correlation of PO-Bado-SF GB with self-reporting instruments

The PO-Bado-SF GB showed a better discrimination between patients in distress and those not in distress (DT ≥6) at a cut-off of 4.5 (AUC 0.806, sensitivity: 72.2% specificity: 72.8%) than in patients in need or not in need of treatment according to their HSI results (cut-off: 3.5, AUC: 0.749, sensitivity: 81.7% specificity: 56.4%). Sensitivity for correct discrimination was moderate with the highest sensitivity and specificity at 6 months for both variables. Further information on the results of the ROC-analysis can be seen in Table 4 and Supplementary Table 3). A large correlation was found between Po-Bado SF GB and DT. Furthermore all, but one PO-Bado SF single item (“other issues”) showed a large correlation with the PO-Bado-SF GB (Table 5).

**Table 5:** Correlation analysis: PO-Bado GB vs. total score and subscores, PO-Bado vs. DT, PO-Bado GB and total score vs. EORTC GHS

	Correlation-coefficient	*p-*value
***PO-Bado GB/***Po-Bado total score	0.73	<0.0001
***PO-Bado GB/***fatigue	0.53	<0.0001
***PO-Bado GB/***mood swings	0.6	<0.0001
***PO-Bado GB/***fear	0.65	<0.0001
***PO-Bado GB/***depression	0.64	<0.0001
***PO-Bado GB/***functional limitations	0.53	<0.0001
***PO-Bado GB/***other problems	0.42	<0.0001
***DT***PO-Bado GB	0.650	<0.0001
***DT/***PO-Bado total score	0.6	<0.0001
***EORTC GHS***/PO-Bado GB	−0.555	<0.0001
***EORTC GHS***/PO-Bado total score	−0.551	<0.0001

### PO-Bado scores in correlation to hrQoL

Median patient reported GHS ranged between 50 (t1) and 75 at t3 (range 0–100). Detailed EORTC results are displayed in Supplementary Table 4.

A large correlation was found between PO-Bado-SF total score and GB, as well as between both general PO-Bado Scores and DT and EORTC QLQ-C30 GHS, respectively (Table 5). The detailed results of the correlation analysis between PO-Bado total score and GB and the EORTC QLQ-C30/BN20 subscales are displayed in the supplementary (Supplementary Table 5). In summary, large correlations were seen between PO-Bado total score and the following EORTC subscales: GHS, physical functioning, emotional functioning, social functioning, fatigue, future uncertainty and drowsiness, as well as between PO-Bado GB and EORTC Subscale for GHS and emotional functioning.

## DISCUSSION

In our study we applied the PO-Bado SF in brain tumor patients during the early disease trajectory after first diagnosis. We evaluated the interviewer-based expert rating for distress screening in cancer patients longitudinally in a sample of 140 patients. Our analyses revealed that a relevant proportion (41–47%, t1–t3) of brain tumor patients reached a total score > 8 (indicating significant burden [15, 17] and that the PO-Bado SF results were only partially in accordance to the results of the self-reporting instruments DT and HSI.

### Results of PO-Bado SF and self-reporting instruments

Mean time of assessment was 7.5 min (range 5–18.5 min) during the study. In some cases due to the mentioned questions the patient-doctor consultation was prolonged. This has to be taken into account regarding feasibility in clinical routine. Further, the application was in a patient subgroup fitting into the study inclusion criteria what leads to a certain selection bias. However, also when implementing PRO measures in order to screen for distress the indicated problems or items mentioned by the patients have to be discussed what can prolong patient-doctor consultation. Finally, the implementation of ClinRO has to be proven in clinical practice, although we feel that in brain tumor patients they may be useful.

In line with the results of Marten-Mittags, the item with the lowest mean was “other problems” and the item with the highest mean was “anxiety/worries/tension” postoperatively. However, after three and six months “fatigue/tiredness” became of greatest importance. This emphasizes that fatigue plays a major role for brain tumor patients, which is reported also by patients themselves in up to 90% [21–23].

The mean total score of PO-Bado SF in our study was comparable (mean of t1-t3: 7.89) to others [17–19]. The high proportion of patients scoring ≥8 at all three measurements indicates the high burden, which is also reflected in the DT score and HSI results. Although DT scores improved over time after first diagnoses, the proportion of patients in need for psychosocial help according to HSI remained stable. However, to our knowledge, a minimal important difference (MID) for DT scores has not been analyzed so far, it remains difficult to interpret if these improvements were also clinically relevant. Additionally, the two instruments were only in 54–62% of the screenings in agreement. This shows that the two screening instruments assess different aspects of psychosocial burden: The HSI seems to be more specific with regard to psycho-oncological support, whereas the DT assess general burden, what has been shown by our group in a former study [24]. Similarly, the PO-Bado SF GB was higher at t1 and decreasing to t3 (t1: 4.32, t3: 3.75). This may reflect the shock and burden after first being confronted with the diagnosis [25]. Even though total scores decreased in 15% of patients below the cut-off of 8, most of them remained either above or below this cut-off value in line with the initial postoperative scoring. The majority of patients with high scores postoperatively on the PO-Bado SF total score and a complete follow up continued to score above 8 at later assessments. Therefore the decrease seen in the PO-Bado SF GB may be a reflection of patients with greater burden dropping out. However, more patients with total scores above 8 completed all assessments compared to those dropping out over time (29% vs. 18%), indicating a possible mix of effects.

Interestingly, the PO-Bado SF total score showed the highest peak at t2 with high item means in “fatigue/tiredness”, “function” and “anxiety/worries”. As also reported by others, patients with brain tumors, who undergo radiation after surgery, show high symptom burden right after the treatment, which improves over time [26, 27]. However, not all patients underwent further therapy, and therefore, other reasons for the perceived fatigue should be taken into account: for instance patients may realize after several months that symptoms after surgery improve but do not subside completely leading to a higher perception of fatigue and loss of function. So far no longitudinal study has been conducted applying the PO-Bado SF in patients with intracranial tumors, thus we are not able to compare the results with others. However, they seem to be in line with the self-reporting of neuro-oncological patients reported in our former studies as well as by others [1, 2, 24, 28].

Additionally to the different screening measures, we investigated the relation between PO-Bado SF and the EORTC QLQ-C30/BN20, because we feel that distress and perceived health related QoL are closely linked, what has been shown in previous studies [3, 26, 29, 30]. However, changes in HRQoL may be perceived differently by patients, care-givers and physicians. We observed in a previous study, that physical function showed only a moderate correlation with distress scores [30]. While the correlation between DT and physical functioning was of moderate strength in this cohort, the PO-Bado SF total score and GB showed large correlations with the physical functioning subscale emphasizing the assumption that the physician’s perception (ClinRO) differs from the patient’s view (PRO).

Further, GHS, emotional functioning and future uncertainty showed large correlations with PO-Bado total score and GB, whereas other functioning scores (role functioning, cognitive function) demonstrated only moderate correlations with both PO-Bado, what may be explained by the heterogeneous patient cohort [30]. Interestingly, the correlation between fatigue and PO-Bado total score was larger compared to the correlation with the PO-Bado GB indicating that total scores may be superior to single items. These heterogeneous results underline the need for the use of different measures to comprehensively assess patients’ distress and HRQol.

### Cutoffs in brain tumor patients

Psychosocial screening instruments–either self-reporting (PRO measures) or expert-rating scales (ClinRO) – are developed in order to divide patients into groups either in or not in need of psychosocial support, thus cutoff scores are required. With regard to PO-Bado SF total score, cutoff scores >8 and >9 were reported: Marten-Mittag found with their cutoff >9, that 36% of their heterogeneous patient sample suffering from clinically relevant distress [17, 18]. Our cutoff elaborated by applying the HSI as reference instrument was 9.5 whereas the cutoff by using the DT was 8.5 what is comparable to the reported results and shows that the cutoffs elaborated for cancer patients can be applied in brain tumor patients as well. The specificity and sensibility were lower than those reported by others. This may be caused by the heterogeneous patient sample. However, the sensitivity in DT was higher with a lower specificity in line with the higher specificity and lower sensitivity of the cutoffs based on the HSI as the two screening instruments measure different aspects of burden. Interestingly, the PO-Bado SF GB showed high concordance with the PO-Bado SF total score, indicating that the global burden perceived by the physicians reflects in a way the total score as a result of the single items. We therefore calculated cutoff scores for the GB as well. In daily routine, although patient reported outcomes and psychosocial screening become more and more important, the psychosocial assessments are not always implementable due to several reasons [8, 11, 31]. Furthermore, patients are not always able to fill in paper-pencil or electronic questionnaires. In these situations, our results indicate that a GB estimated by the physician could reliably indicate a need for psychosocial support. Off note, the physicians’ perception might not always reflect the patients view as shown by our agreement analysis and thus screening procedures should not be replaced by the estimation of the GB by the physician. But we would attribute the GB a certain signaling function, supported by the cutoff score and the correlation between GB and total score.

### Applying an expert rating scale in brain tumor patients

Usually, psychosocial screening is performed by applying short self-reporting questionnaires or screening instruments. However, the topics patients most require help with may not be always the topics that physicians or other health care professional feel necessary or even able to address [31]. This underlines that the patients’ view can differ from physicians’ view and – as recently shown by others – from the caregivers’ view [32], meaning at the same time, that psychosocial treatment needs expressed by patients and the need perceived by experts may be different. However, unlike some reports for estimation of patients’ distress by experts using single-item assessments or visual analogue scales [33–35], the PO-Bado SF is an expert rating based on a short interview. Therefore it not only allows an assessment, but also can raise physicians’ attention for patients’ problems leading to an improved patient-doctor relationship. By assessing several items, the physician allows distressed patients to talk about their problems probably also leading to some relief, thus providing some sort of treatment while screening [15, 16]. Additionally, in this investigation a large correlation was seen between the subjective rating of distress (DT) and the physician’s perception (PO-Bado total score and GB). Thus, this interview may aid in overcoming the gap between patients’ and physicians’ view at least in some areas of life.

Finally, taken into account the critical aspects of external assessment, the PO-Bado SF turned out to be implementable in the consultation in our study. Even if the evaluation of the full length interview during daily routine is not always feasible, using the global burden (GB) as indicator if psychosocial distress is present seems to be reliable, as we have found a large correlation between the SF total score and physician assessed global burden (GB). Optimally the assessment comprises both: self-reporting instruments and external assessment. Finally, a low-threshold offer of psycho-oncological interventions should be available and the provision of psychosocial support should not be based solely on self-reporting or external assessment in brain tumor patients.

### Limitations and strengths of the study

Off note, our study has some limitations: due to the inclusion criteria, we have to assume a certain selection bias as only patients were included being able to fill in questionnaires. Second, we included patients with first diagnosis of any intracranial tumor, leading to a heterogeneous patient sample with primary and secondary, malignant and benign tumors. At the same time, it is one of the strength of the study, as the complete spectrum of patients in neuro-oncological units is reflected by the results. Third, several interviewers (*n =* 3) with difference in their clinical experience conducted and rated the PO-Bado SF, which may have influenced the ratings, but again the results are therefore more applicable to daily routine with physicians at different levels of training treating and screening patients. As the interviewers of PO-Bado were also the treating physicians, they were aware of the patients’ history, prognosis and further therapies, which might also have biased the evaluation. Moreover, we observed a high drop out of the study, partially due to death indicating the severity of the disorder. However, in observational studies assessing patient reported outcomes, drop-outs are frequently [36], as patients may decline repeated assessments or not further be able to respond the questionnaires.

However, we were able to follow the patients for the first six months along their disease trajectory and the PO-Bado SF total score cutoffs turned out to be stable during the three measurements. Finally, at the same time we conducted external assessment and self-reporting psychosocial screening as well as evaluation of health-related quality of life leading to get a holistic impression of our patient sample.

## MATERIALS AND METHODS

### Study setting and participants

Patients with first diagnosis of a benign or malignant, primary or secondary brain tumor at one of two neurosurgical departments in Germany (department 1 = University Medical Center, department 2 = Community Hospital) were recruited after surgery and histopathological confirmation of the tumor entity as shown in Figure 1. Inclusion criteria were a) first diagnosis of an intracranial tumor of any histology, b) age >18 years, c) absence of significant postsurgical neurocognitive deficits, evaluated by a neurological examination including a Mini Mental Status Test (MMST, score >26/30), or dysphasia, d) given informed consent.

Informed consent was obtained after surgery by (MR, AKH and MNO). Each patient was screened three times for psychosocial burden, general health perception, health-related quality of life (HRQoL) and distress using the NCCN Distress Thermometer [9] (DT), the Hornheide Screening Instrument [37, 38] (HSI), the European Organization for Research and Treatment of Cancer Quality of Life Questionnaire with its brain cancer module (EORTC QLQ-C30 + EORTC QLQ-BN20 [39, 40]) and the Basic Documentation for Psycho- Oncology Short Form (PO-Bado SF [18]). The physicians (MR, MNO, AKH) using the PO-Bado SF were trained according to the manual. Further patients were asked if at all three time points if systematically they wished to receive psychosocial support because of their subjectively perceived burden regardless of their screening results.

Screenings were performed postoperatively during the inpatient period at day 5–7 after surgery once the patient had been informed about the tumor’s entity, as well as at three and six months postoperatively during the routine outpatient visits. In case of benign neoplasms the third assessment was performed over the phone and questionnaires to be filled in by the patients were sent by mail free of charge for each patient. When sending the questionnaires by mail, patients had to return them within 2 weeks. If not they were reminded by the study members by a phone call. When questionnaires were not returned within 4 weeks, patients were excluded from the study.

Additionally, demographic and tumor specific data (entity, location, extend of resection) and pre- as well as postoperative Karnofsky Performance Scale (KPS) scores were collected. A flow chart of the study design is provided in Figure 1.

### Applied instruments and questionnaires

The Basic Documentation for Psycho- Oncology Short Form (PO-Bado SF) is an external assessment tool for oncologist developed from the PO-Bado standard version [15, 17] in order to assess patients’ somatic and psychological burden based on a structured interview. The short form consists of 6 single items: “fatigue/tiredness”; “mood swings/helplessness/vulnerability”; “anxiety/worries/tension”; “depression/ grief”; “functional limitations in daily activities”, “other problems” (e.g., social or family problems)’. Each item applies a Likert Scale reaching from 0 (no burden) to 4 (great burden) based on a 5 to 10 min face-to-face-interview, which can be conducted by professional health personnel in inpatient or outpatient situations. Additionally the interviewer assess the global burden (GB) on an analogue scale ranging from 0–10 with higher numbers indicating greater burden during the last 3 days as perceived by the professional. A manual and an interview guideline were provided by the authors with instructions for the PO-Bado short form (http://www.po-bado.med.tum.de/). The clinical cutoff criteria indicating need for psychooncological intervention proposed by Herschbach et al. are the following: a) at least one of the six PO-Bado SF items is scored 4, b) two items are scored 3. Alternatively, a PO-Bado SF total score of ≥8 is defined as the second cutoff criterion [15, 17]. In our project, we applied the cut-off score ≥8 indicating significant burden to assess agreement between measures (DT, HSI).

The National Comprehensive Cancer Network (NCCN) Distress Thermometer (DT) measures distress of cancer patients in terms of a single item presented as an 11-point visual analogue scale with scores from 0 (not distressed) to 10 (extremely distressed) along with further 40 items concerning financial, physical, emotional and spiritual concerns [9, 41–43]. In brain tumor patients, a score of 6 or above on the numeric rating scale (NRS) is recommended as a cut-off score for a clinically significant level of distress [1, 9]. The DT is widely used in cancer centers for screening.

The European Organization for Research and Treatment of Cancer Quality of life core Questionnaire (EORTC QLQ-C30) is a frequently used patient reported outcome measure including 30 items to assess health-related quality of life in oncological patients in terms of functioning and symptom burden [39]. The items form five functional scales (physical, role, emotional, social and cognitive functioning), three symptom scales (fatigue, nausea and vomiting, pain), six single-item scales (dyspnea, insomnia, appetite loss, constipation, diarrhea and financial difficulties) and one global health status/QoL scale. The brain module (QLQ-BN20) contains 20 items developed for brain tumor patients, comprising four functional scales, of which three neurological deficit scales and one future uncertainty scale, as well as seven single items for treatment- and disease-related symptoms [39, 40, 44].

The Hornheide Screening Instrument (HSI) was initially applied to evaluate psychosocial burden of skin tumor patients and developed from the Hornheide questionnaire [38, 45]. The HSI has been proven to be as valid and reliable as the more extensive questionnaire. For the clinical application, the HSI uses a cut-off score of >4. Respectively, a discriminant analysis tool, established and evaluated by Strittmatter *et al.*, is provided in order to screen patients in need of psychosocial support [45, 46]. Although it has not been validated specifically in brain tumor patients, the instrument is recommended in Germany by the German Cancer Society for screening cancer patients in clinical routine and has also been applied by Fischbeck *et al.* and our group recently in brain tumor patients [24, 47].

### Statistical analysis and outcomes

Primary outcome was correlation between PO-Bado SF total score as well as PO-Bado SF GB and self-reporting instruments (DT, HSI, EORTC) and to evaluate the PO-Bado SF total score and GB cutoff for brain tumor patients based on DT and HSI with respective sensitivity and specificity.

Secondary outcomes were results of the PO-Bado-SF assessment at t1-t3 in patients with intracranial tumors and agreement between external assessment und self-reporting questionnaires concerning need for support/clinically relevant distress. Agreement in PO-Bado SF with self-reporting questionnaires was defined as follows:

PO-Bado SF with DT: agreement +/+ = Patients with PO-Bado SF total score ≥ 8 and DT ≥ 6, agreement −/− = patients with PO-Bado SF total score <8 and DT<6.

PO-Bado SF with HSI: Agreement +/+ = PO-Bado SF total score ≥8 and patient in need of psycho-oncological support according to the discriminant analysis tool as well as agreement −/− = PO-Bado SF total score <8 and patient in not need of psycho-oncological support according to the discriminant analysis tool.

Cohen’s Kappa statistics were performed to assess agreement. κ = 0.01–0.2 was defined as slight, κ = 0.21–0.4 as fair, κ = 0.41–0.60 as moderate, κ = 0.61–0.80 as substantial and κ = 0.81–0.99 as almost perfect agreement [20].

Demographic and tumor-related data as well as KPS were analyzed descriptively. Differences in distribution between study centers were estimated using Mann-Whitney-*U*-Test or Pearson-Chi-Square test as appropriate based on the measurement level.

Results from the questionnaires were tested for normal distribution using the Kolmogorov-Smirnov-Test. Pearson or Spearman-Rho-Correlations between DT-scores/EORTC functioning and symptom scores and PO-Bado scores were used as appropriate according to the data’s distribution. Correlations >0.5 were described as large, >0.3 as moderate and >0.1 as small [48]. ANOVA with post-hoc Gabriel’s pairwise test was applied to estimate differences in PO-Bado SF GB Scores, total scores and DT scores between tumor entities.

Sensitivities and specificities for PO-Bado GB and total score cut-off values based on results of the HSI and DT scores were determined using ROC-Curves. Cut-offs were selected based on the optimal balance between sensitivity and specificity [49].

For the ROC- and correlations analyses all assessment were assumed to be independent samples and therefore cumulated, because the current state of psychosocial burden was assessed at each assessment using different screening tools evaluating their agreement and no specific psycho-oncological treatment was initiated in between possibly influencing the following screening. Similarly, Martin-Mittag *et al.* cumulated their data of patients at different time points [18].

*P* < 0.05 was considered statistically significant. All analyses were performed using SPSS, version 18.0 (IBM Corp., North Castle, NY, USA).

### Ethics

This study was performed in accordance with the Helsinki Declaration after approval by the local ethics committee (No. 837.220.12 (8321-f)). The responsible clinical investigators (MR, AKH and MNO) informed eligible patients verbally and handed out written information about the study. Participants provided their written informed consent and were assigned a patient identifier to ensure data confidentiality.

## CONCLUSIONS

The physicians’ perception by the PO-Bado-SF provides a different aspect of psychosocial burden patients with intracranial tumors and cutoffs for brain tumor patients could be elaborated in the study. However, the physicians’ view does not completely reflect the patients’ wishes. Therefore, patient reported outcome measures are indispensable, but should be accompanied by the assessment of the physicians.

## SUPPLEMENTARY MATERIALS TABLES






