# Supplementary material for: Screening for distress in patients with intracranial tumors during the first 6 months after diagnosis using self-reporting instruments and an expert rating scale (the basic documentation for psycho-oncology short form – PO-Bado SF)
Source: Oncotarget. 2018 Jul 24;9(57):31133–45. doi: 10.18632/oncotarget.25763 (PMC6089557; doi:10.18632/oncotarget.25763)
Supplement: Supplementary file 1 [file oncotarget-09-31133-s001.pdf]

Screening for distress in patients with intracranial tumors during the first 6 months after diagnosis using self-reporting instruments and an expert rating scale (the basic documentation for psycho-oncology short form – PO-Bado SF)

SUPPLEMENTARY MATERIALS

Supplementary Table 1: PO-Bado results over time

PO-Bado results over time in relation to follow-up and to t1

a) Change in PO-Bado in relation to PO-Bado total score at t1		
	n	%
remained < 8	38	26.6
decreased < 8	22	15.4
increased ≥ 8	26	18.2
remained ≥ 8	52	36.4
missing	5	3.5
b) Dropouts		
	n	%
dropout after ≥ 8 postop	26	18.2
dropout after <8 postop	15	10.5
complete FU after ≥ 8 postop	42	29.4
complete FU after <8 postop	46	32.2
other		
missing	10	7
c) Change in Po-Bado in relation to dropout		
dropout after ≥ 8 postop	*n*	%
remained ≥ 8	23	88.5
decreased <8	3	11.5
dropout after <8 postop	n	%
remained<8	9	60
increased ≥ 8	6	40
complete FU ≥ 8 postop	n	%
remained ≥ 8	26	61.9
decreased <8	16	38.1
complete FU <8 postop	n	%
remained <8	25	54.3
increased ≥ 8	20	43.5
decreased <8	1	2.2

Supplementary Table 2: Results of subpopulations according to tumor entity

	Metastasis	Glioma (WHO I-IV)	Meningioma	Other	*p*-value
PO-Bado GB postop (mean ± SD)	4.5 ± 1.5	4.8 ± 2.1	3.8 ± 2.0	3.5 ± 1.5	0.026
PO-Bado GB 3 months (mean ± SD)	3.8 ± 2.0	4.4 ± 2.3	4.0 ± 2.6	3.1 ± 2.5	0.380
PO-Bado GB 6 months (mean ± SD)	4.3 ± 2.2	4.3 ± 2.4	3.2 ± 2.5	3.0 ± 3.1	0.176
PO-Bado total score postop (mean ± SD)	7.9 ± 2.2	8.6 ± 4.2	6.6 ± 3.9	6.4 ± 3.7	0.073
PO-Bado total score 3 months (mean ± SD)*	9.5 ± 5.3	9.3 ± 5.5	7.9 ± 5.3	4.4 ± 3.8	0.025
PO-Bado total score 6 months (mean ± SD)	8.6 ± 5.3	8.8 ± 5.5	6.5 ± 5.7	5.5 ± 4.8	0.172
DT postop (mean ± SD)	4.5 ± 1.9	5.7 ±2.6	4.4 ± 2.5	4.2 ± 2.1	0.033
DT 3 months (mean ± SD)	3.7 ± 2.0	4.5 ± 2.7	3.9 ± 2.8	3.8 ± 2.6	0.559
DT6 months (mean ± SD)	3.6 ± 2.1	4.0 ± 2.2	3.4 ± 3.0	2.6 ± 2.4	0.569

*post hoc analysis: metastasis vs. other $p = 0.047$; astrocytic tumor vs. other $p = 0.02$.
All pairwise comparisons $p > 0.05$.

Supplementary Table 3: Detailed results of ROC analyses. See Supplementary_Table_3

Supplementary Table 4: Detailed EORTC results t1-t3

	median (range) Overall	median (range) postop	median (range) @ 3 months	median (range) @ 6 months
GHS	58.3 (0–100)	50 (0–100)	58.3 (0–100)	75 (0–100)
Physical Functioning	80 (0–100)	80 (26.7–100)	80 (0–100)	86.7 (0–100)
Role Functioning	66.7 (0–100)	66.7 (0–100)	66.7 (0–100)	83.3 (0–100)
Emotional Functioning	66.7 (0–100	58.3 (0–100)	75.0 (0–100)	83.3 (0–100)
Cognitive Functioning	83.3 (0–100)	66.7 (0–100)	83.3 (0–100)	83.3 (0–100)
Social Functioning	66.7 (0–100)	66.7 (0–100)	66.7 (0–100)	83.3 (0–100)
Fatigue	33.3 (0–100)	33.3 (0–100)	33.3 (0–100)	33.3 (0–100)
Nausea & Vomiting	0.0 (0–83.3)	0.0 (0–83.3)	0.0 (0–66.7)	0.0 (0–66.7)
Pain	0.0 (0–100)	16.7 (0–100)	0.0 (0–100)	0.0 (0–83.3)
Insomnia	33.3 (0–100)	33.3 (0–100)	33.3 (0–100)	33.3 (0–100)
Appetite Loss	0.0 (0–100)	0.0 (0–100)	0.0 (0–100)	0.0 (0–100)
Constipation	0.0 (0–100)	0.0 (0–100)	0.0 (0–100)	0.0 (0–100)
Diarrhoea	0.0 (0–66.7)	0.0 (0–66.7)	0.0 (0–66.7)	0.0 (0–66.7)
Financial Difficulties	0.0 (0–100)	0.0 (0–100)	0.0 (0–100)	0.0 (0–100)
Future Uncertainty	29.2 (0–100)	41.7 (0–100)	25.0 (0–100)	16.7 (0–100)
Visual Disorder	0.0 (0–100)	11.1 (0–100)	0.0 (0–100)	0.0 (0–100)
Motor Dysfunction	11.1 (0–100)	11.1 (0–100)	11.1 (0–88.9)	0.0 (0–100)
Communication Deficit	0.0 (0–100)	11.1 (0–100)	0.0 (0–88.9)	0.0 (0–100)
Headache	33.3 (0–100)	33.3 (0–100)	0.0 (0–100)	0.0 (0–100)
Seizure	0.0 (0–100)	0.0 (0–100)	0.0. (0–100)	0.0 (0–100)
Drowsiness	33.3 (0–100)	33.3 (0–100)	33.3 (0–100)	33.3 (0–100)
Itchy Skin	0.0 (0–100)	0.0 (0–100)	0.0 (0–100)	0.0 (0–100)
Hair Loss	0.0 (0–100)	0.0 (0–100)	0.0 (0–100)	0.0 (0–100)
Weakness of Legs	0.0 (0–100)	0.0 (0–100)	0.0 (0–100)	0.0 (0–100)
Bladder Control	0.0 (0–100)	0.0 (0–100)	0.0 (0–100)	0.0 (0–100)

Supplementary Table 5: Correlation between PO-Bado and EORTC instrument. See Supplementary_Table_5
