# Supplementary material for: Screening for distress in patients with intracranial tumors during the first 6 months after diagnosis using self-reporting instruments and an expert rating scale (the basic documentation for psycho-oncology short form – PO-Bado SF)
Source: Oncotarget. 2018 Jul 24;9(57):31133–45. doi: 10.18632/oncotarget.25763 (PMC6089557; doi:10.18632/oncotarget.25763)
Supplement: Supplementary file 2 [file oncotarget-09-31133-s002.docx]

**Supplementary Table 3: Detailed results of ROC analyses**

median (range) median (range) median (range) median (range)

Overall postop @ 3 months @ 6 months

GHS 58.3 (0-100) 50 (0-100) 58.3 (0-100) 75 (0-100)

Physical Functioning 80 (0-100) 80 (26.7-100) 80 (0-100) 86.7 (0-100)

Role Functioning 66.7 (0-100) 66.7 (0-100) 66.7 (0-100) 83.3 (0-100)

Emotional Functioning 66.7 (0-100) 58.3 (0-100) 75.0 (0-100) 83.3 (0-100)

Cognitive Functioning 83.3 (0-100) 66.7 (0-100) 83.3 (0-100) 83.3 (0-100)

Social Functioning 66.7 (0-100) 66.7 (0-100) 66.7 (0-100) 83.3 (0-100)

Fatigue 33.3 (0-100) 33.3 (0-100) 33.3 (0-100) 33.3 (0-100)

Nausea & Vomiting 0.0 (0-83.3) 0.0 (0-83.3) 0.0 (0-66.7) 0.0 (0-66.7)

Pain 0.0 (0-100) 16.7 (0-100) 0.0 (0-100) 0.0 (0-83.3)

Insomnia 33.3 (0-100) 33.3 (0-100) 33.3 (0-100) 33.3 (0-100)

Appetite Loss 0.0 (0-100) 0.0 (0-100) 0.0 (0-100) 0.0 (0-100)

Constipation 0.0 (0-100) 0.0 (0-100) 0.0 (0-100) 0.0 (0-100)

Diarrhoea 0.0 (0-66.7) 0.0 (0-66.7) 0.0 (0-66.7) 0.0 (0-66.7)

Financial Difficulties 0.0 (0-100) 0.0 (0-100) 0.0 (0-100) 0.0 (0-100)

Future Uncertainty 29.2 (0-100) 41.7 (0-100) 25.0 (0-100) 16.7 (0-100)

Visual Disorder 0.0 (0-100) 11.1 (0-100) 0.0 (0-100) 0.0 (0-100)

Motor Dysfunction 11.1 (0-100) 11.1 (0-100) 11.1 (0-88.9) 0.0 (0-100)

Communication Deficit 0.0 (0-100) 11.1 (0-100) 0.0 (0-88.9) 0.0 (0-100)

Headache 33.3 (0-100) 33.3 (0-100) 0.0 (0-100) 0.0 (0-100)

Seizure 0.0 (0-100) 0.0 (0-100) 0.0. (0-100) 0.0 (0-100)

Drowsiness 33.3 (0-100) 33.3 (0-100) 33.3 (0-100) 33.3 (0-100)

Itchy Skin 0.0 (0-100) 0.0 (0-100) 0.0 (0-100) 0.0 (0-100)

Hair Loss 0.0 (0-100) 0.0 (0-100) 0.0 (0-100) 0.0 (0-100)

Weakness of Legs 0.0 (0-100) 0.0 (0-100) 0.0 (0-100) 0.0 (0-100)

Bladder Control 0.0 (0-100) 0.0 (0-100) 0.0 (0-100) 0.0 (0-100)
