# Supplementary material for: Screening for distress in patients with intracranial tumors during the first 6 months after diagnosis using self-reporting instruments and an expert rating scale (the basic documentation for psycho-oncology short form – PO-Bado SF)
Source: Oncotarget. 2018 Jul 24;9(57):31133–45. doi: 10.18632/oncotarget.25763 (PMC6089557; doi:10.18632/oncotarget.25763)
Supplement: Supplementary file 3 [file oncotarget-09-31133-s003.docx]

**Supplementary Table 5:** **Correlation between PO-Bado and EORTC instrument**

***correlation-coefficient p-value***

**PO-Bado SF total score vs.**

GHS -0.55 <0.0001

Physical Functioning -0.51 <0.0001

Role Functioning -0.48 <0.0001

Emotional Functioning -0.55 <0.0001

Cognitive Functioning -0.46 <0.0001

Social Functioning -0.51 <0.0001

Fatigue 0.53 <0.0001

Nausea & Vomiting 0.18 0.002

Pain 0.41 <0.0001

Insomnia 0.38 <0.0001

Appetite Loss 0.21 <0.0001

Constipation 0.19 0.001

Diarrhoea 0.14 0.012

Financial Difficulties 0.28 <0.0001

Future Uncertainty 0.56 <0.0001

Visual Disorder 0.30 <0.0001

Motor Dysfunction 0.47 <0.0001

Communication Deficit 0.32 <0.0001

Headache 0.32 <0.0001

Seizure 0.17 0.003

Drowsiness 0.50 <0.0001

Itchy Skin 0.31 <0.0001

Hair Loss 0.26 <0.0001

Weakness of Legs 0.40 <0.0001

Bladder Control 0.20 <0.0001

**PO-Bado SF GB vs.**

GHS -0.56 <0.0001

Physical Functioning -0.50 <0.0001

Role Functioning -0.43 <0.0001

Emotional Functioning -0.51 <0.0001

Cognitive Functioning -0.42 <0.0001

Social Functioning -0.45 <0.0001

Fatigue 0.49 <0.0001

Nausea & Vomiting 0.15 0.006

Pain 0.43 <0.0001

Insomnia 0.36 <0.0001

Appetite Loss 0.23 <0.0001

Constipation 0.17 0.002

Diarrhoea 0.17 0.003

Financial Difficulties 0.28 <0.0001

Future Uncertainty 0.54 <0.0001

Visual Disorder 0.28 <0.0001

Motor Dysfunction 0.39 <0.0001

Communication Deficit 0.25 <0.0001

Headache 0.33 <0.0001

Seizure 0.16 0.004

Drowsiness 0.44 <0.0001

Itchy Skin 0.23 <0.0001

Hair Loss 0.22 <0.0001

Weakness of Legs 0.35 <0.0001

Bladder Control 0.18 0.001

**DT vs.**

GHS -0.52 <0.0001

Physical Functioning -0.47 <0.0001

Role Functioning -0.45 <0.0001

Emotional Functioning -0.60 <0.0001

Cognitive Functioning -0.46 <0.0001

Social Functioning -0.44 <0.0001

Fatigue 0.49 <0.0001

Future Uncertainty 0.54 <0.0001
